# Suicidal Ideation and Suicide Completion in Benzodiazepine Users: A Systematic Review of Current Evidence

**DOI:** 10.7759/cureus.84318

**Published:** 2025-05-18

**Authors:** Safiyyah M Khan, Alousious Kasagga, Anushka Verma, Eiman Saraya, Mehjabin S Haque, Mithum Senaratne, Safeera Khan

**Affiliations:** 1 Medicine, Basaveshwara Medical College and Hospital, Chitradurga, IND; 2 Pathology, Peking University, Beijing, CHN; 3 Internal Medicine, Smt. Nathiba Hargovandas Lakhmichand (NHL) Municipal Medical College, Ahmedabad, IND; 4 Psychiatry, St. Martinus University, Willemstad, CUW; 5 Neurology, California Institute of Behavioral Neurosciences and Psychology (CIBNP), Fairfield, USA; 6 Internal Medicine, California Institute of Behavioral Neurosciences and Psychology (CIBNP), Fairfield, USA; 7 Family Medicine, Michener Institute at University Health Network (UHN), Toronto, CAN

**Keywords:** benzodiazepines, drug safety, monitoring, overdose, suicide

## Abstract

Benzodiazepines (BZDs) are widely used anxiolytics for treating various psychiatric conditions and for procedures requiring conscious sedation. Despite their therapeutic benefits, there is concern about their paradoxical effects, particularly the potential increase in suicidal ideation and suicide completion. This systematic review examines the extent to which benzodiazepines contribute to, cause, or exacerbate suicidal ideation and suicide completion. Adhering to the Preferred Reporting Items for Systematic Reviews and Meta-Analyses (PRISMA) 2020 guidelines, we conducted a systematic review of literature from databases including PubMed, PubMed Central (PMC), and PubPsych. Search terms related to benzodiazepines and suicide were used, yielding 7,961 articles. After removing duplicates and applying inclusion and exclusion criteria, 587 articles were screened, leading to a final selection of six articles. These studies underwent rigorous quality assessment using various tools. The review highlighted several key findings. Moderate benzodiazepine use with concomitant psychotherapy or antidepressants was associated with reduced suicide risk. Concurrent use of opioids and benzodiazepines significantly elevated the risk of suicide attempts and intentional self-harm. Benzodiazepine use was linked to increased suicide risk in vulnerable groups, including those with pre-existing mental health conditions. Benzodiazepines should be prescribed with caution, ideally for short-term use until antidepressant effects manifest. Close monitoring for addiction, withdrawal, and suicidal ideation is essential. Prescribers must be aware of the increased risks when benzodiazepines are used in conjunction with opioids or in patients with heightened vulnerability to suicide. Effective weaning programs and risk assessment tools are crucial to mitigate these risks and ensure patient safety.

## Introduction and background

Benzodiazepines (BZDs) are anxiolytics currently employed in the treatment of a wide variety of psychiatric illnesses as well as various procedures such as conscious sedation. They serve as a pharmacological cornerstone in the management of anxiety and sleep disorders and are frequently used as adjunctive therapy in the treatment of schizophrenia and other psychotic disorders. Although evidence from recent literature suggests that benzodiazepines may increase the overall risk of suicide attempts or completions [1], the underlying mechanisms, particularly paradoxical effects such as increased suicidal ideation, remain insufficiently understood. Further research is needed to clarify causality and inform future prescribing practices. Benzodiazepines can offer short-term relief from anxiety, but they are often associated with a paradoxical increase in anxiety over the long term [2]. A Scandinavian nationwide case-crossover study showed a significantly increased risk of suicidal behavior following a benzodiazepine prescription [3], which leads one to wonder if the risk can be extrapolated to other countries worldwide. This review is guided by the Population, Intervention, Comparison, Outcome (PICO) framework. The population includes adults exposed to benzodiazepines compared to those who are not, and the outcome being measured is suicidal ideation, attempt, or completion.

## Review

Methodology

This systematic review was conducted using the Preferred Reporting Items for Systematic Reviews and Meta-Analyses (PRISMA) 2020 guidelines [4]. A formal meta-analysis was not conducted due to considerable heterogeneity across studies in terms of design (case-control, cross-sectional, and case report), benzodiazepine type and dosage, outcome definitions (e.g., suicidal ideation versus attempts versus completions), and population characteristics. As a result, a narrative synthesis was deemed more appropriate to capture the nuances of the available evidence.

Search Sources and Strategy

We searched medical databases such as PubMed, PubMed Central (PMC), and PubPsych for relevant medical literature. The identified papers were screened, eligibility criteria were applied, and 10 research papers were identified. The finalized papers explored the role of benzodiazepines in directly causing or worsening suicidal ideation.

We conducted searches across all databases using different combinations of benzodiazepines and suicide keywords. Specifically, in PubMed, in addition to these keywords, we devised and applied the following strategy to search pertinent literature in PubMed's MeSH database: (((“Benzodiazepines”[MeSH]) AND (“suicide”[MeSH]))). The databases utilized and the corresponding numbers of identified papers for each database are outlined in Table 1.

**Table 1:** Databases utilized and the corresponding numbers of identified papers for each database MeSH: Medical Subject Headings

Keywords/search strategy	Database used	Number of results
(("Benzodiazepines"[MeSH]) AND ("suicide"[MeSH]))	PubMed MeSH database	145
Benzodiazepines AND suicide	PubMed	151
Benzodiazepines AND suicide	PubPsych	253
Benzodiazepines AND suicide	PMC	7,412
Total research papers identified	All databases	7,961
Number of articles after removing duplicates	All databases	587

Inclusion and Exclusion Criteria

We chose articles published within the last five years (2017-2022). Our selection was limited to papers written in English or those for which a complete English translation of the text was accessible. Any articles for which we could not obtain the full text were excluded. Additionally, we did not consider gray literature or proposal papers for inclusion. Trials with incomplete or unclear outcome reporting were excluded.

Selection Process

We moved the selected articles to EndNote and eliminated any duplicate papers. Each article underwent screening based on its title and abstract and was individually reviewed by SMK (primary author). If there was any disagreement regarding eligibility, these issues were deliberated with all co-authors and resolved through a collective agreement. The chosen articles were then subjected to a comprehensive assessment of their full texts, with only pertinent articles being considered. We applied specific criteria for inclusion and exclusion, resulting in the final list of articles that met these criteria.

Quality Assessment

We assessed the quality of the selected articles using appropriate tools for quality appraisal. All co-authors participated in the quality assessment process. The quality assessment of the included studies underscores their overall methodological rigor while also identifying potential sources of bias. The majority of studies exhibited a low risk in key areas, such as randomization procedures, adherence to interventions, outcome measurement, and result reporting. The overall risk of bias was deemed low for most studies, reinforcing the reliability of the evidence synthesized in this review.

Data Collection Process

Once the articles were selected for the systematic review and gathered, the main results and essential details were examined. Data extraction was carried out independently by SK and AK, and all authors participated equally in refining the collected data and interpreting the outcomes as indicated by the data extraction surveys.

Results

Study Identification and Selection

After evaluating these papers by reviewing titles and summaries and obtaining the complete texts, 46 articles were chosen as potential candidates. These selected full-text articles underwent evaluation for suitability and quality, resulting in the finalization of seven articles for the purpose of review. Post selection, one article was retracted and therefore excluded from this study, resulting in six articles for review.

**Figure 1:**
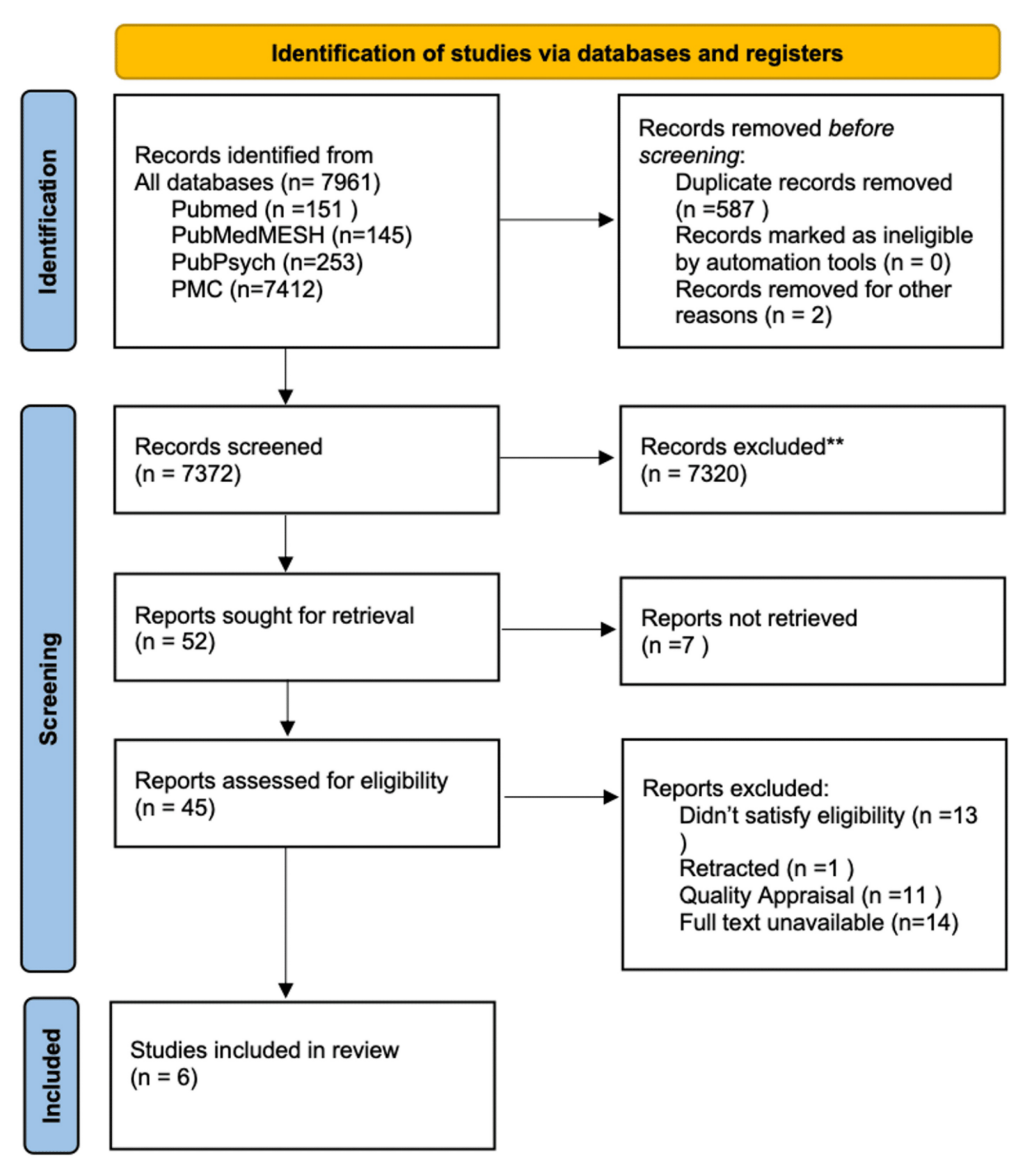
PRISMA flowchart showing the process of article selection PRISMA: Preferred Reporting Items for Systematic Reviews and Meta-Analyses, PMC: PubMed Central

The process of selecting the studies is visually presented in Figure 1 of the PRISMA flowchart.

The eligibility of the articles was evaluated using appropriate quality assessment instruments. The outcomes of the quality appraisal are presented in Tables 2-4.

**Table 2:** Quality appraisal for case-control studies

Case-control studies	Type of study	Points awarded	Maximum possible points
Boggs et al. (2019) [5]	Retrospective case-control study	8	11
Cato et al. (2019) [6]	Prospective case-control study	8	11
Gibbons et al. (2021) [7]	Prospective case-control study	9	11

**Table 3:** Quality appraisal for cross-sectional studies JBI: Joanna Briggs Institute

Cross-sectional studies	Tool used for quality assessment	Result
Ghosh et al. (2020) [8]	Quality Assessment Tool for Observational Cohort and Cross-Sectional Studies	Fair
Reid Finlayson et al. (2022) [9]	JBI checklist for cross-sectional studies	5/8

**Table 4:** Quality appraisal for case reports JBI: Joanna Briggs Institute

Case report	Tool used for quality assessment	Points awarded	Maximum points possible
Mustonen et al. (2021) [10]	JBI critical appraisal checklist for case reports	6	8

Outcomes Measured

The primary outcomes extracted from the finalized research papers were the role of benzodiazepines in causing or worsening suicidal ideation or suicide completion. The secondary outcomes assessed were whether the role of benzodiazepines was worsened or improved with concomitant prescriptions and therapies.

Study Characteristics

We reviewed six research papers with a total of 4,774,225 patients. Out of these finalized studies, three were case-control studies [5-7], two were cross-sectional studies [8,9], and one was a case report [10].

All studies involved patients prescribed benzodiazepines. Worsening or new suicidal ideation or suicide completion was reported in most studies.

Table 5 shows a summary and characteristics of all included studies.

**Table 5:** Study characteristics OXP: oxazepam

Author and year of publication	Type of study	Purpose of the study	Number of participants	Results	Conclusions
Boggs et al. (2019) [5]	Retrospective case-control study	To evaluate the association between suicide death and concordance with benzodiazepine guidelines	6,960	Benzodiazepine guideline concordance was associated with reduced odds for suicide in patients with anxiety disorders and was driven by shorter duration of benzodiazepine use with concomitant psychotherapy or antidepressant medication.	They found reduced odds for suicide in those with anxiety disorders who filled benzodiazepines in short-moderate duration with concomitant psychotherapy or antidepressant treatment.
Cato et al. (2019) [6]	Prospective case-control study	To test the hypothesis that benzodiazepines are associated with an increased risk of suicide	154	Benzodiazepine prescriptions were more common among cases than controls.	These data indicate that benzodiazepine use may increase the risk of suicide.
Ghosh et al. (2020) [8]	Cross-sectional study	To determine the epidemiology and prevalence of recent benzodiazepine exposure among suicide deaths in Colorado from 2015 to 2017	3,465	Among the 3,465 suicide deaths in Colorado between 2015 and 2017, 20% had recent benzodiazepine exposure.	Benzodiazepines have been linked to suicidal ideation, but population-level assessments of benzodiazepine exposure among suicide deaths are rare.
Gibbons et al. (2021) [7]	Prospective case-control study	To examine whether prescription opioids and benzodiazepines interact to increase the rate of suicide attempts and intentional self-harm	4,762,438	There was a larger association for benzodiazepines with suicide attempt, intentional self-harm, and drug overdose.	Increased risk of suicide attempt, intentional self-harm, and drug overdose for concomitant use of opioids and benzodiazepines is in large part attributable to benzodiazepine use alone.
Mustonen et al. (2021) [10]	Case report	To describe a unique case of high-dose OXP withdrawal	1	Tapering off high doses of benzodiazepines is possible within a relatively short time period in a hospital setting.	Discontinuing very high-dose benzodiazepines is achievable.
Reid Finlayson et al. (2022) [9]	Cross-sectional study	To assess the experiences of those taking, tapering, or having discontinued benzodiazepines	1,207	Suicidal thoughts or attempted suicide were reported by 54.4%.	Greater awareness is needed for both prescribers and patients about the potential for a difficult withdrawal from benzodiazepines.

Discussion

Each year, approximately 30.6 million adults (12.6% of the US population) report using benzodiazepines. Among them, 25.3 million (10.4%) use the medication as prescribed, while 5.3 million (2.2%) engage in misuse [11]. One study highlighted significant increases in mortality, including but not limited to suicide attempts, among patients on stable long-term benzodiazepine therapy who discontinue treatment compared to those who continue [12]. Another indicated that benzodiazepines should be prescribed cautiously for patients with borderline personality disorder (BPD), as they are linked to a higher risk of suicide [13]. Neutel et al. found that the link between benzodiazepine use and attempted suicide is particularly strong among young individuals, men, and those not using antidepressants [14]. Among high-dose regular benzodiazepine users, the prevalence of a history of suicide attempts was significantly higher in those with comorbid BPD than in those without [15]. One study reinforced previous findings associating alprazolam with a higher risk of suicide attempts, stating that the elevated risk applies to benzodiazepines as a whole, regardless of their half-life or potential for withdrawal seizures [16].

An assessment of the risk of bias in the chosen articles is shown in Table 6.

**Table 6:** Risk of bias assessment ROBINS-I: Risk Of Bias In Non-randomised Studies of Interventions, EMR: electronic medical record, NOS: Newcastle-Ottawa Scale, DiD: difference-in-differences, JBI: Joanna Briggs Institute

Study (author and year)	Design	Risk of bias tool used	Domains assessed	Overall risk of bias	Notes
Boggs et al. (2019) [5]	Retrospective case-control	ROBINS-I	Confounding, selection, misclassification, bias in reporting	Moderate	Large EMR dataset, matching used, possible residual confounding
Cato et al. (2019) [6]	Matched case-control	NOS	Selection, comparability, exposure	Moderate	Matched on age, sex, and diagnosis; adjusted for prior suicide attempts; potential indication bias
Gibbons et al. (2021) [7]	Pharmacoepidemiologic cohort	ROBINS-I	Confounding, selection, misclassification, bias in reporting	Low	Large sample, used inverse probability weighting and the DiD model, strong design
Ghosh et al. (2020) [8]	Population-level epidemiologic assessment	Adapted ROBINS-I	Exposure assessment, outcome ascertainment, missing data	Moderate	Uses death certificates and toxicology; strong exposure definition, limited by observational nature
Reid Finlayson et al. (2022) [9]	Cross-sectional online survey	AXIS tool	Sampling, measurement bias, non-response bias	High	Voluntary self-report online survey; high risk of recall and selection bias
Mustonen et al. (2021) [10]	Case report	JBI checklist for case reports	Patient history, intervention clarity, follow-up, outcome reporting	High	Single patient, not generalizable; clinical detail is high but lacks comparison

Benzodiazepine Exposure: Duration, Recency, and Amount

Boggs et al. indicated that the safest duration of benzodiazepine use across the entire sample was 1-2 fills, whereas higher quantities were associated with an increased risk of suicide [5]. The National Institute for Health and Care Excellence (NICE) indicates that benzodiazepines may be effective in handling short-term crises and during initial treatment before antidepressants have reached therapeutic benefit [17]. Boggs et al. also found that the association of suicide risk was significantly reduced in individuals with moderate benzodiazepine use (3-8 fills) when concomitant antidepressant or psychotherapy treatments were added [5].

Ghosh et al. found that nearly a third of those who died by suicide had either filled a benzodiazepine prescription in the past three years or tested positive for benzodiazepines post-mortem. Of these, 14% had a prescription within 30 days of death. When considering both prescriptions within 30 days and positive toxicology, recent benzodiazepine exposure rose to 20% of suicide deaths [8].

Benzodiazepine Dispensing Appeared Higher in the 30-Day Risk Period

This indicates that current and recent benzodiazepine use is riskier than past use. The duration may exceed two bottle fills as long as another antidepressant or other therapy is being maintained. BZDs must not form the mainstay of treatment for any psychiatric disorder and must be used for a short period.

Recent benzodiazepine use (within the last 30 days) was found to be significantly linked to hospitalization for suicide or suicide attempts compared to past use. This association was observed in both patients with and without recent psychiatric history, with a particularly strong effect observed in those without such a history.

We conclude from the above observations that if benzodiazepines must be prescribed, they must be to tide over the patient in the initial short-term phase before the antidepressant effect has fully set in, following which it is ideal to discontinue them owing to the significant suicide risk associated with exposure to them. Benzodiazepines should be prescribed as a gateway to complete recovery and must not be continued indefinitely owing to the suicide risk, especially in psychiatric patients. Patients on benzodiazepines must be closely monitored for addiction and withdrawal, as well as screened for suicidal tendencies and warned of them. Prescribers and patients must both be made aware of the higher percentage of suicidal deaths as well as suicidal ideation seen in patients on benzodiazepines, and carefully and individually tailored programs to wean patients off benzodiazepines must be developed. In addition, a questionnaire to assess each patient's risk would be ideal.

Risks of Co-administration With Opioids

Gibbons et al. established that concurrent treatment with both opioids and benzodiazepines was linked to a higher risk of suicide attempts and intentional self-harm compared to using either drug alone or neither drug [7].

While the risks of self-harm with BZDs alone seem established, their association when concurrently used with opioids should be underscored. Prescribers must be made aware of the additional risks associated when BZDs are co-administered with opioids.

Prescribing Benzodiazepines in Vulnerable Patients

According to Cato et al., the link between benzodiazepines (BZDs) and suicide might be due to the fact that BZDs are often prescribed to individuals already at a higher risk of suicide due to their anxiety and insomnia symptoms (indication bias). Alternatively, BZDs themselves might causally elevate the risk of suicide [6].

Reid Finlayson et al. revealed that 54% of respondents experienced suicidal thoughts while taking, tapering, or after discontinuing benzodiazepines. Benzodiazepines are linked to an increased risk of suicide or suicide attempts, making this high proportion particularly concerning [9].

Boggs et al. highlighted that future research should investigate the benefits of suicide risk screening in patients with anxiety disorders who are treated with benzodiazepines, especially those under the care of primary care providers (PCPs). PCPs, who prescribed 55% of benzodiazepines in the USA, are twice as likely as psychiatrists to prescribe these medications in conflict with treatment guidelines, particularly in monotherapy for anxiety disorders [5].

This underscores the importance of selectively prescribing benzodiazepines according to the likelihood of the patient developing or succumbing to suicidal ideation. An objective scoring system could be developed to assess a patient's risk, and it can be used to determine if the patient should be continued on a BZD at future visits.

Patients Who Have Developed Addiction or Tolerance

The case report by Mustonen et al. indicated that it is possible to taper off extremely high doses of benzodiazepines within a relatively short time in a hospital setting. In this case, they used antiepileptics, α-2-agonists, and long-acting benzodiazepines. They emphasize that sustaining abstinence necessitates close cooperation between inpatient and outpatient treatment facilities. Physicians should proactively inform patients about the risks of abruptly discontinuing high-dose benzodiazepine medication, including life-threatening complications [10].

This case report demonstrated that even if a patient has been prescribed benzodiazepines for too long or has been taking too much, safe and effective weaning is possible with close monitoring.

Interpretation of Findings

The results of this systematic review indicate a significant association between benzodiazepine use and increased suicidal ideation and behavior. This finding is consistent with previous studies suggesting that while benzodiazepines can effectively manage acute anxiety, their long-term use is fraught with risks. The observed paradoxical effects, such as increased suicidal ideation, may stem from the neurochemical changes induced by prolonged benzodiazepine use. These changes can potentially exacerbate underlying psychiatric conditions, leading to adverse outcomes.

Among the six included studies, several demonstrated statistically significant associations between benzodiazepine use and suicide risk. In a matched case-control study by Cato et al., individuals who died by suicide were significantly more likely to have been prescribed benzodiazepines than matched controls (42.2% versus 27.9%; odds ratio (OR) = 1.89, 95% confidence interval (CI): 1.17-3.03, p = 0.009), and this remained significant after adjustment (OR = 1.83, 95% CI: 1.06-3.14) [6].

A large cohort analysis found a robust association between benzodiazepines and suicide attempt or self-harm (hazard ratio (HR) = 2.55, 95% CI: 2.12-3.05), while concomitant use with opioids had a subadditive interaction (HR = 0.70, 95% CI: 0.55-0.89), potentially reflecting a complex interplay of risk factors in polypharmacy [7].

State-level epidemiologic data from Colorado (2015-2017) revealed that 20% of suicide decedents had recent benzodiazepine exposure, with notably higher prevalence among women (34%) than men (16%). Benzodiazepine exposure was most common in suicides by drug overdose (48%), suggesting a method-specific association [8].

Another study examining treatment guidelines found that short- to moderate-term benzodiazepine use in conjunction with psychotherapy or antidepressants was associated with lower odds of suicide among individuals with anxiety disorders (OR = 0.611, 95% CI: 0.392-0.953, p = 0.03). No significant protective effect was found for those with sleep disorders (p = 0.08) [5].

Complementing these quantitative studies, a large-scale qualitative survey of individuals who used or discontinued benzodiazepines revealed that 54.4% experienced suicidal thoughts or attempted suicide, alongside a wide spectrum of debilitating symptoms such as insomnia, anxiety, cognitive and balance issues, and gastrointestinal distress. Social, occupational, and recreational functioning were severely impacted, with 86.3% reporting problems in friendships and 46.8% losing employment. Most respondents (76.2%) had not been informed about the risks of long-term use or withdrawal [9].

Collectively, the findings underscore a nuanced risk profile: while inappropriate or prolonged benzodiazepine use may elevate suicide risk, structured, guideline-concordant usage may mitigate it. Furthermore, the high prevalence of suicidality in user reports highlights the need for improved patient education and monitoring during both initiation and discontinuation of benzodiazepines.

Comparison With Existing Literature

Our findings align with existing literature that highlights the risks associated with benzodiazepine use, particularly in vulnerable populations. Additionally, our review corroborates findings from other studies that suggest the concomitant use of benzodiazepines and opioids significantly elevates the risk of suicide attempts and intentional self-harm.

Mechanisms Behind Paradoxical Effects

The mechanisms underlying the paradoxical effects of benzodiazepines are not fully understood but may involve alterations in GABAergic neurotransmission. Chronic benzodiazepine use can lead to changes in GABA receptor sensitivity, potentially resulting in reduced efficacy of inhibitory neurotransmission and heightened anxiety or agitation [18]. This neurochemical imbalance might contribute to increased suicidal ideation, particularly during withdrawal periods when the brain is readjusting to the absence of the drug.

Clinical Implications

Given the significant risks identified, it is crucial for healthcare providers to exercise caution when prescribing benzodiazepines. Short-term use, accompanied by close monitoring, should be the standard approach. For patients requiring longer-term anxiety management, alternative treatments such as selective serotonin reuptake inhibitors (SSRIs) or cognitive-behavioral therapy (CBT) should be prioritized. Furthermore, prescribers must be vigilant when benzodiazepines are used in combination with opioids, given the compounded risk of adverse outcomes.

Risk Assessment and Monitoring

The development and implementation of risk assessment tools can aid in identifying patients at higher risk of suicidal ideation when prescribed benzodiazepines. Regular follow-up and monitoring are essential to detect early signs of paradoxical reactions. Tailored weaning programs should be established to help patients discontinue benzodiazepines safely, thereby reducing the risk of withdrawal symptoms and subsequent suicidal behavior.

Future Research Directions

Future research should focus on longitudinal studies to better understand the long-term effects of benzodiazepine use on suicidal ideation and behavior. Investigating the neurobiological mechanisms underlying paradoxical reactions can provide insights into safer prescribing practices. Moreover, developing standardized guidelines for benzodiazepine discontinuation, particularly in populations at high risk of misuse and addiction, is essential. Studies exploring the effectiveness of risk assessment tools and monitoring strategies in clinical practice would also be beneficial.

In conclusion, while benzodiazepines remain a valuable tool for managing acute anxiety, their potential to induce suicidal ideation necessitates cautious and informed prescribing. By prioritizing short-term use, implementing rigorous monitoring protocols, and exploring alternative treatments, healthcare providers can mitigate the risks associated with benzodiazepine use. Ongoing research and the development of evidence-based guidelines will be crucial in optimizing patient outcomes and ensuring the safe use of these medications.

Limitations

The review faces several limitations that hinder the ability to draw consistent conclusions about the relationship between benzodiazepine use and suicidal ideation. Variability in how studies defined and reported benzodiazepine dosage and duration leads to inconsistencies in findings. Many studies relied on retrospective data, which is subject to recall bias and incomplete records, affecting the accuracy of the results. Self-reporting of suicidal ideation and benzodiazepine use may be prone to underreporting or misreporting, introducing potential inaccuracies. Misclassification of exposure or outcomes, such as off-label benzodiazepine use, could bias the results. One limitation of this review is that many studies did not specify which benzodiazepine was used in the treatment regimen, nor did they provide detailed dosage information. As a result, the review could not assess the effects of individual drugs within the class. Future studies with more detailed reporting on the specific drugs used and their respective dosages will allow for more targeted analysis and comparison. Additionally, the review lacks comprehensive longitudinal data needed to understand the long-term effects of benzodiazepine use. Differences in diagnostic criteria for psychiatric disorders across studies impact the consistency and comparability of findings. Unmeasured confounders, such as socioeconomic factors or comorbid substance use, may not have been adequately accounted for, potentially influencing the results. The included studies varied in design and quality, with some being case reports or cross-sectional studies that do not provide as robust evidence as randomized controlled trials. This variation in quality affects the reliability and generalizability of the conclusions.

## Conclusions

This systematic review delineates the complex association between benzodiazepine use and suicidal ideation, highlighting significant risks inherent in both therapeutic and non-therapeutic contexts. While short-term, moderate benzodiazepine use, particularly when integrated with antidepressants or psychotherapy, can be beneficial, chronic use, high dosages, recent exposure, and concurrent opioid use substantially heighten the risk of suicide. Certain populations, including chronic users, individuals with borderline personality disorder, children, and those with neurological or learning disorders, appear especially vulnerable to the paradoxical effects of these medications, necessitating careful clinical consideration.

Accordingly, prescribing benzodiazepines demands a cautious, judicious approach, with an emphasis on short-term application while longer-term therapies are established. Regular monitoring, comprehensive patient education, and individualized weaning protocols are critical to minimizing dependency and mitigating withdrawal risks. Future research should prioritize the development of objective risk stratification tools and deepen understanding of the mechanisms underlying paradoxical reactions. Through more rigorous prescribing practices and vigilant patient management, clinicians can better balance the therapeutic utility of benzodiazepines against their potential for harm, thereby enhancing patient outcomes and safety.
